# Evaluation of an IoT Strain-Sensing System with LoRa Telemetry and Asset Metadata

**DOI:** 10.3390/s26185686

**Published:** 2026-09-08

**Authors:** Xiaoxiao Bu, Shenshi Jiang, Henry Gong, Tommy Chaung, Enbang Li, Zhengyi Jiang

**Affiliations:** 1 School of Engineering, University of Wollongong, Wollongong, NSW 2522, Australia; xb376@uowmail.edu.au; 2 School of Mathematics and Physics, University of Wollongong, Wollongong, NSW 2522, Australia; sj863@uowmail.edu.au; 3 Roobuck Pty Ltd., Brookvale, NSW 2100, Australia; henryg@roobuck.com.au (H.G.); tommyc@roobuck.com.au (T.C.)

**Keywords:** strain sensing, temperature compensation, edge processing, history recovery, LoRa, instrumented rock bolt, asset metadata, Internet of Things (IoT)

## Abstract

Instrumented rock-bolt monitoring requires strain records that retain integrity and asset context after local processing and wireless transmission. An Internet of Things (IoT) strain-sensing system with LoRa telemetry and asset metadata was evaluated on a cantilever fixture. The node converted tare-referenced bridge counts to apparent strain, applied temperature compensation using a lagged temperature term, evaluated alarms locally, and buffered summaries for LoRa transfer. Histories were assessed using sequence continuity and a 32-bit cyclic redundancy check (CRC-32). Ten monotonic loading runs showed linear responses to nominal screw advance (*R*^2^ > 0.9997); fitted slopes had a coefficient of variation of 0.55%. Across three fixed-setting tests spanning 5.1–8.0 °C, unchanged embedded compensation reduced the magnitude of fitted apparent thermal sensitivity by 89.1–95.5%. In all 45 operator-controlled alarm trials, trigger or non-trigger outcomes matched expectations recorded before dashboard inspection. Of 60 planned application-layer history transfers, 50 passed; the remaining ten comprised four failures, three interruptions, and three invalid or contaminated trials. Passed transfers included exact reconstruction of a 1024-record circular buffer. Retrieved metadata passed CRC-32 verification and matched the registered laboratory asset. These results characterize a one-asset laboratory workflow before packaged-bolt, underground, and multi-asset validation.

## 1. Introduction

Rock bolts stabilize underground excavations by transferring load between unstable strata and the surrounding stable rock mass [1,2]. Monitoring changes in rock-bolt response is therefore important for assessing the condition of installed ground support over time [2,3,4]. Existing monitoring approaches use either distributed or discrete sensing. Distributed optical-fiber systems resolve strain variation along the installed length [5,6,7], whereas bonded electrical-resistance gauges [2,3,8] and fiber Bragg grating (FBG) sensors [4] measure changes at selected sections. For selected monitoring locations, discrete Internet of Things (IoT) sensing offers a practical architecture in which measurements can be processed locally and transmitted wirelessly to a surface monitoring system.

For discrete systems based on bonded electrical-resistance strain gauges, a central measurement challenge is separating mechanically induced response from temperature-related output. Temperature-dependent resistance and differential thermal expansion among the gauge, adhesive, and substrate can produce apparent strain even when the nominal mechanical setting is unchanged [9,10]. During heating or cooling, temperature gradients between the gauge and substrate can introduce additional transient error [11]. Temperature compensation under transient conditions should therefore account for both quasi-steady temperature dependence and thermal lag.

Wireless telemetry adds a second challenge. In underground roadways, link quality varies with tunnel geometry, antenna placement, physical obstructions, multipath fading, and line-of-sight availability [12,13,14,15]. Because wireless capacity and node energy are limited, edge nodes commonly process samples locally and transmit summaries or selected features rather than continuous raw-sample streams [16,17,18,19,20,21].

Packaged rock-bolt sensing [3], temperature compensation [10], and low-power edge processing or telemetry [19,21] have generally been studied as separate functions. Less attention has been given to how sensing, compensation, alarms, wireless transfer, history recovery, and asset-metadata matching operate together within one discrete monitoring system.

This gap matters operationally because a useful monitoring record must preserve more than sensor output. Surface users need a temperature-compensated trend, evidence of local alarms, a way to retrieve recent stored records when records are missing, and enough asset information to identify the support that produced the data. A sensor may respond correctly yet still leave the surface user with an incomplete record or uncertainty about the event or support to which the data belong. Monitoring records are used to assess support condition [4]; losing event or asset context therefore reduces their operational value. Linking measurements to persistent asset and location metadata can also support future digital-twin applications [22,23,24,25,26].

This study evaluates an integrated IoT strain-sensing system using a controlled cantilever fixture. The laboratory tests examine fixture-level measurement repeatability, embedded temperature compensation under natural temperature variation, local alarm execution, routine LoRa telemetry, history reconstruction after controlled application-layer faults, and metadata matching for a registered asset. The study therefore focuses on the integration and laboratory evaluation of these functions within a common monitoring workflow. Absolute strain calibration, packaged-bolt mechanical response, underground radio performance, and multi-asset operation remain subjects for subsequent validation.

The remainder of this paper is organized as follows. Section 2 describes the intended operational role and system architecture, local signal processing and temperature compensation, alarm logic, communication and recovery mechanisms, and laboratory evaluation methods. Section 3 presents the repeated-loading, fixed-setting natural-temperature, routine-telemetry, alarm, history-transfer, and metadata-matching results. Section 4 discusses the measurement interpretation, operational significance, communication recovery, metadata matching, limitations, and applicability to underground deployment. Section 5 summarizes the conclusions and outlines the required bolt-scale, underground, and multi-asset validation.

## 2. Materials and Methods

### 2.1. Intended Operational Role and System Scope

In the intended deployment, each edge node would monitor a selected instrumented rock bolt. The intended users are geotechnical engineers and mine operations staff, who would receive temperature-compensated apparent-strain summaries, alarm states, on-demand access to recent stored measurements, and the bolt’s identification and location metadata. These outputs allow a surface user to view the current response, examine the records surrounding an alarm, and confirm which support produced the data.

The node performs tare-referenced apparent-strain conversion, temperature compensation, and alarm checks locally. The surface application logs incoming records, reconstructs requested history, and compares returned metadata with a registry. Local processing reduces the need to transmit every raw sample or wait for the surface application before detecting an alarm.

**Figure 1:**
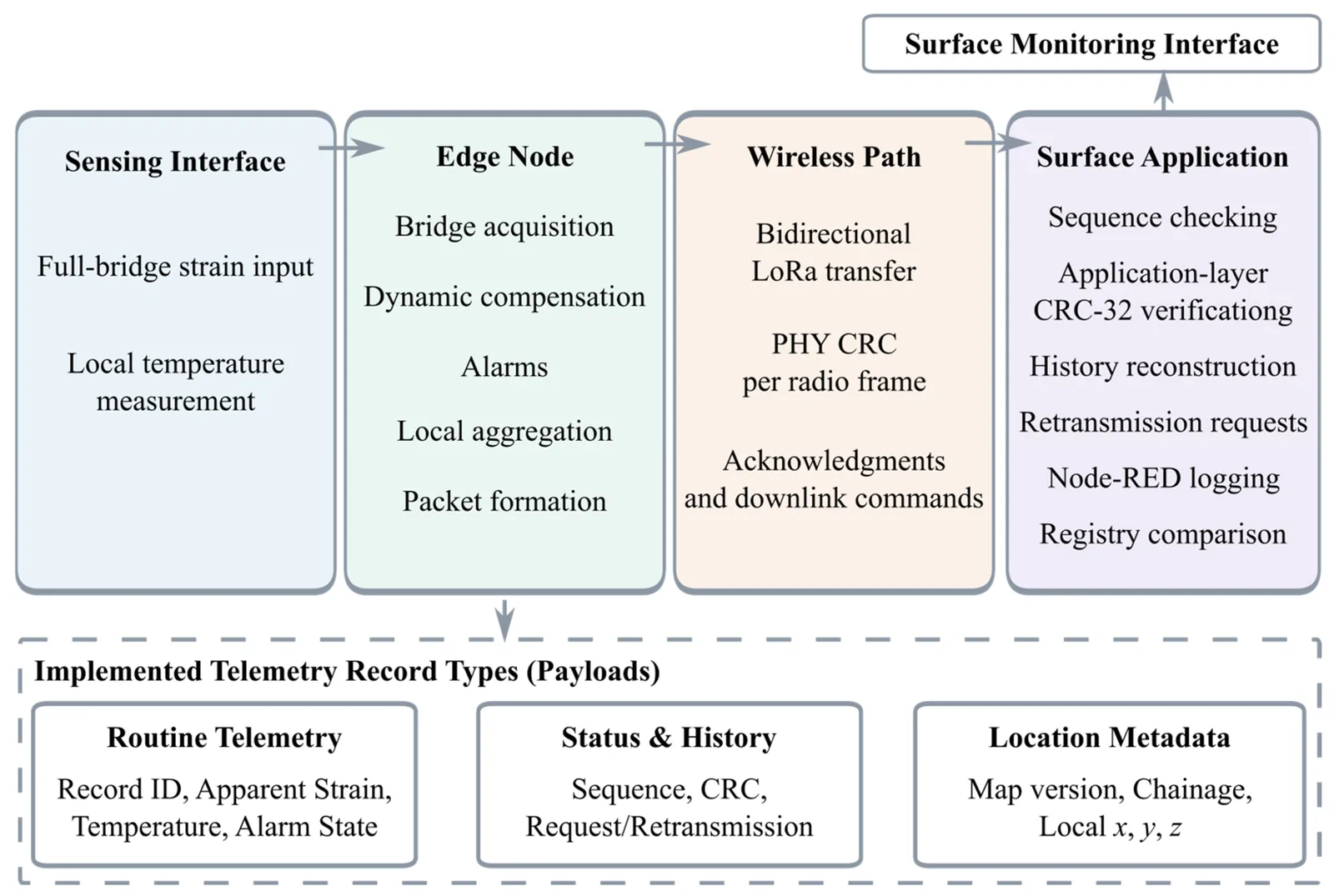
Data path from local bridge and temperature acquisition to LoRa–MQTT transfer, surface record handling, and asset-metadata comparison. Abbreviations: CRC, cyclic redundancy check; MQTT, Message Queuing Telemetry Transport; PHY, physical layer.

Figure 1 shows the data path. The sensing interface acquired each bridge measurement together with a local temperature reading. The edge node processed valid samples, assembled routine and event records, and transmitted them over a bidirectional LoRa link to the Message Queuing Telemetry Transport (MQTT) bridge. Downlink commands controlled telemetry and requested stored data. The bridge relayed payloads over MQTT to the Node-RED surface application, where history and location payloads underwent a 32-bit cyclic redundancy check (CRC-32) verification before decoding and registry comparison. Matched records were then displayed in the surface monitoring interface.

The link carried three record classes (Figure 1). Routine telemetry records contained 1 s measurement summaries and alarm-state transitions. Status and history records managed requests, carried sequenced copies of buffered summaries, and reported transfer completion. When the surface application detected a missing sequence, it could request retransmission and reconstruct the specified interval. Location-metadata records contained the fixture identifier, map version, coordinate reference, installation details, and configuration identifiers, allowing the received stream to be compared with the corresponding laboratory registry. History-recovery fault injection was applied after the wireless transport stage, within the Node-RED application stream. The principal hardware comprised a BF1000-3EB 1000 Ω full-bridge foil strain gauge marked “Made in China”, Adafruit TMP117 QT and NAU7802 QT boards (Product IDs 4821 and 4538; Adafruit Industries, LLC, Brooklyn, NY, USA), and an RAK3172 module (Shenzhen RAKwireless Technology Co., Ltd., Shenzhen, China). The surface gateway used a custom CM4-based LoRa repeater (Roobuck Pty Ltd., Brookvale, NSW, Australia) incorporating a Raspberry Pi Compute Module 4 Rev. 1.1 with 1 GB RAM (Raspberry Pi Ltd., Cambridge, UK). The strain-gauge manufacturer could not be identified. Table 1 summarizes the components, software, and tested settings.

### 2.2. Signal Acquisition and Local Record Formation

Bridge conversions were performed at 10 Hz, and only completed, non-saturated samples were retained. Each valid bridge sample was paired with a temperature measurement for apparent-strain conversion, temperature compensation, and alarm evaluation. Approximately ten processed samples were aggregated into each 1 s routine record. These procedures follow established strain-gauge conversion and windowed edge-processing methods [9,10,16,19,20,21].

For an accepted sample i, the tare-referenced uncompensated apparent strain $\varepsilon_{i}$ was calculated as follows:

(1) $$\varepsilon_{i}=k_{s}\left(c_{i}-C_{0}\right)$$

where $c_{i}$ is the digitized bridge count, $C_{0}$ is the tare count established separately at the start of each session, and $k_{s}$ is the fixed count-to-apparent-strain coefficient (µε/count).

The archived metadata record a session-specific geometry-based coefficient associated with a nominal 1.0 mm screw advance for each repeated fixture-loading session. The fixed value used in the temperature-compensation and alarm evaluations, $k_{s}=4.31449\times 10^{-4}$ µε/count, matches the archived coefficient for the session corresponding to Run 3 in Supplementary Table S1 and remained unchanged throughout those evaluations. Because no independent strain, displacement, or load reference was used, the converted values are reported as tare-referenced apparent strain rather than absolute strain.

Within reporting window k, the set $W_{k}$ contained $n_{k}$ accepted samples. The mean count and the mean uncompensated and compensated apparent strains were calculated as

(2) $${\bar{y}}_{k}=\frac{1}{n_{k}}\sum_{i\in W_{k}}y_{i},\;y\in \{c,\varepsilon ,\varepsilon_{c}\}.$$

Each routine record included the record identifier, alarm state, latest and lagged temperatures, and aggregated bridge metrics: mean count, within-window standard deviation (SD), and mean uncompensated and compensated apparent strain. The local circular buffer also stored compensated apparent-strain extrema and rate-of-change metrics for later review of events [16,19,20,21].

### 2.3. Embedded Temperature Compensation

Temperature-related strain-gauge output depends on the sensor configuration, and transient temperature gradients can create lag-dependent errors [9,10,11]. The empirical correction used here therefore combines a quasi-steady term referenced to the tare temperature with a transient term based on the difference between measured and lagged temperature.

At sample i, $t_{i}$ denoted sample time, ${\Delta t}_{i}$ represented the elapsed time since the preceding sample, and $T_{i}$ indicated the latest valid temperature. Heating and cooling states were identified from the change in a first-order low-pass-filtered temperature state between successive compensation updates. State transitions used entry and exit thresholds of 0.002 and 0.001 °C, respectively, and required five consecutive updates in the same direction. With time constant *τ*, the update coefficient was

(3) $$\alpha_{i}=1-exp(-\frac{{\Delta t}_{i}}{\tau }).$$

The lagged temperature was then updated as

(4) $$T_{lag,i}=T_{lag,i-1}+\alpha_{i}\left(T_{i}-T_{lag,i-1}\right).$$

The thermal state was quantified using two metrics: the deviation of the lagged temperature from the tare reference temperature ($\Delta T_{ref,i}$) and the short-term transient differential between the measured and lagged temperatures ($\delta T_{i}$):

(5) $$\Delta T_{ref,i}=T_{lag,i}-T_{ref},$$

(6) $$\delta T_{i}=T_{i}-T_{lag,i}.$$

The estimated thermal response $\phi_{i}$ comprised linear and quadratic polynomial terms of the reference-temperature deviation alongside a transient component:

(7) $$\phi_{i}=a_{1}{\Delta T}_{ref,i}+a_{2}{\Delta T}_{ref,i}^{2}+a_{3}{\delta T}_{i}.$$

With $z_{0}$ denoting the zero offset established at tare, the estimated apparent thermal contribution and the compensated apparent strain were calculated as

(8) $$\varepsilon_{T,i}=z_{0}+\phi_{i},$$

(9) $$\varepsilon_{c,i}=\varepsilon_{i}-\varepsilon_{T,i}.$$

The coefficient set was obtained from repeated preliminary fixed-setting sessions and loaded into the firmware before the three reported evaluations. A single coefficient set was used for both heating and cooling branches: $\tau_{heat}$ = $\tau_{cool}$ = 8385.1 s, $a_{1}$ = 2.2921 µε/°C, $a_{2}$ = −0.11650 µε/°C^2^, and $a_{3,heat}$ = $a_{3,cool}$ = 1.3226 µε/°C. Although the firmware recorded heating and cooling states, it did not use branch-specific coefficient values. The coefficients remained unchanged throughout all three evaluations, and none of the evaluation datasets were used for re-estimation. Because the coefficients were identified for this specific sensor–adhesive–cantilever configuration, they require re-estimation or validation for other installations. Supplementary Methods S2 describes the selection process.

Following 200 valid bridge acquisitions, the baseline count $C_{0}$ was defined as their mean. The tare-referenced uncompensated apparent strain supplied to the compensation routine was therefore $\varepsilon_{tare}$ = 0 µε. The firmware then assigned the reference temperature $T_{ref}$ to the concurrent lagged temperature $T_{lag}$, preserving the instantaneous transient differential $T-T_{lag}$. At tare, the initial thermal response $\phi_{tare}=a_{3}(T-T_{lag})$ may be non-zero, yielding the zero offset $z_{0}=\varepsilon_{tare}-\phi_{tare}=-\phi_{tare}$. For Evaluation 1, whose tare occurred within the excluded initial 120 s window, T = 17.258 °C, $T_{lag}$ = 17.203 °C, and $a_{3}$ = 1.3226 µε/°C, giving $z_{0}$ = −0.0727 µε. The same session-specific initialization procedure was applied in Evaluations 2 and 3. The tare temperatures were reported to three decimal places to make the zero-offset calculation transparent. The offset ensured that the compensated apparent strain at tare was zero ($\varepsilon_{c,tare}$ = 0).

### 2.4. Embedded Alarm Rules and Event Formation

Three predefined threshold- and rate-based rules were used to evaluate local alarm execution, consistent with established edge and wireless structural-monitoring approaches [16,17,19,21]. The tests examined whether the node executed the rules, generated and transmitted event records, latched the dashboard state, processed acknowledgments, and cleared alarms under recorded operator-controlled apparent-strain profiles. All strain thresholds in this subsection are expressed on the fixed apparent-strain scale defined in Section 2.2.

The compensated apparent strain was evaluated continuously against three predefined alarm criteria. The upper-limit rule applied a one-sided threshold with 2 µε hysteresis and a one-sample hold. The positive-spike rule compared the current value with a buffered real-time reference sample whose age met the configured reference interval and required both a 10% relative increase and a minimum absolute change of 5 µε.

For the absolute-rate rule, approximately 1 Hz mean apparent-strain values were compared across the configured time window. Defining w as the corresponding record lag and $\Delta t_{i,w}$ as the elapsed time in minutes, the absolute strain rate $r_{i}$ was calculated as

(10) $$r_{i}=\left|\frac{\varepsilon_{c,i}-\varepsilon_{c,i-w}}{\Delta t_{i,w}}\right|.$$

Transitions into or out of an alarm state generated category-specific event records that were queued with priority over routine 1 s summaries.

Alarm thresholds and timing parameters were configured through the Node-RED command interface without firmware recompilation. For example, ALARM SET 10 2 1 0.1 1 configured, in order, a 10 µε upper limit, 2 µε hysteresis, a one-sample hold, a 0.1 (10%) positive-spike criterion, and a 1 s spike-reference interval. Absolute-rate parameters were configured separately. These timing values defined the evaluation windows and historical look-back periods used by the firmware; they were independent of the manually applied loading rate.

### 2.5. Wireless Transfer, Recovery, and Surface Reconstruction

LoRa carried bidirectional messages between the node and gateway [27], and MQTT Version 3.1.1 relayed messages from the gateway to the surface application [28]. At the tested setting, the edge node sent one routine telemetry record per second. High-priority alarm and control messages required acknowledgments, whereas routine telemetry was sent without acknowledgment. History transfers used application-level acknowledgment/negative acknowledgment (ACK/NAK) commands for sequence-based recovery and validation [29], and alarm events required event-specific acknowledgments before clearance. The LoRa physical layer CRC checked individual radio frames, while application-layer CRC-32 checked each reconstructed history or location payload as a whole.

Concurrent with live telemetry, the node retained the latest 1024 summary records in a circular buffer, equivalent to approximately 17 min at the tested nominal 1 Hz reporting rate. In response to a history request, the node divided the requested records into sequenced packets. The surface application produced a reconstructed history CSV only after sequence continuity and CRC checks passed. Supplementary Table S2 details the structural packet layouts, the 32-byte record schema, numeric formats, endianness, and CRC coverage.

After reception, the surface application added reception timestamps and session context to routine and event records before decoding them and matching them with cached asset metadata. A location-metadata packet (LOCATION_V1) contained the fixture identifier, spatial coordinates, installation details, and configuration parameters. After CRC-32 verification, the decoded fields were compared with the registered BOLT-001 fixture in the local coordinate frame. The evaluation covered one registered asset, BOLT-001. Multi-asset registration, reassignment, duplicate-identity handling, and registry scalability require a larger deployment study. Supplementary Table S3 specifies the 78-byte layout and field definitions.

### 2.6. Laboratory Evaluation

#### 2.6.1. Mechanical Loading Test

**Figure 2:**
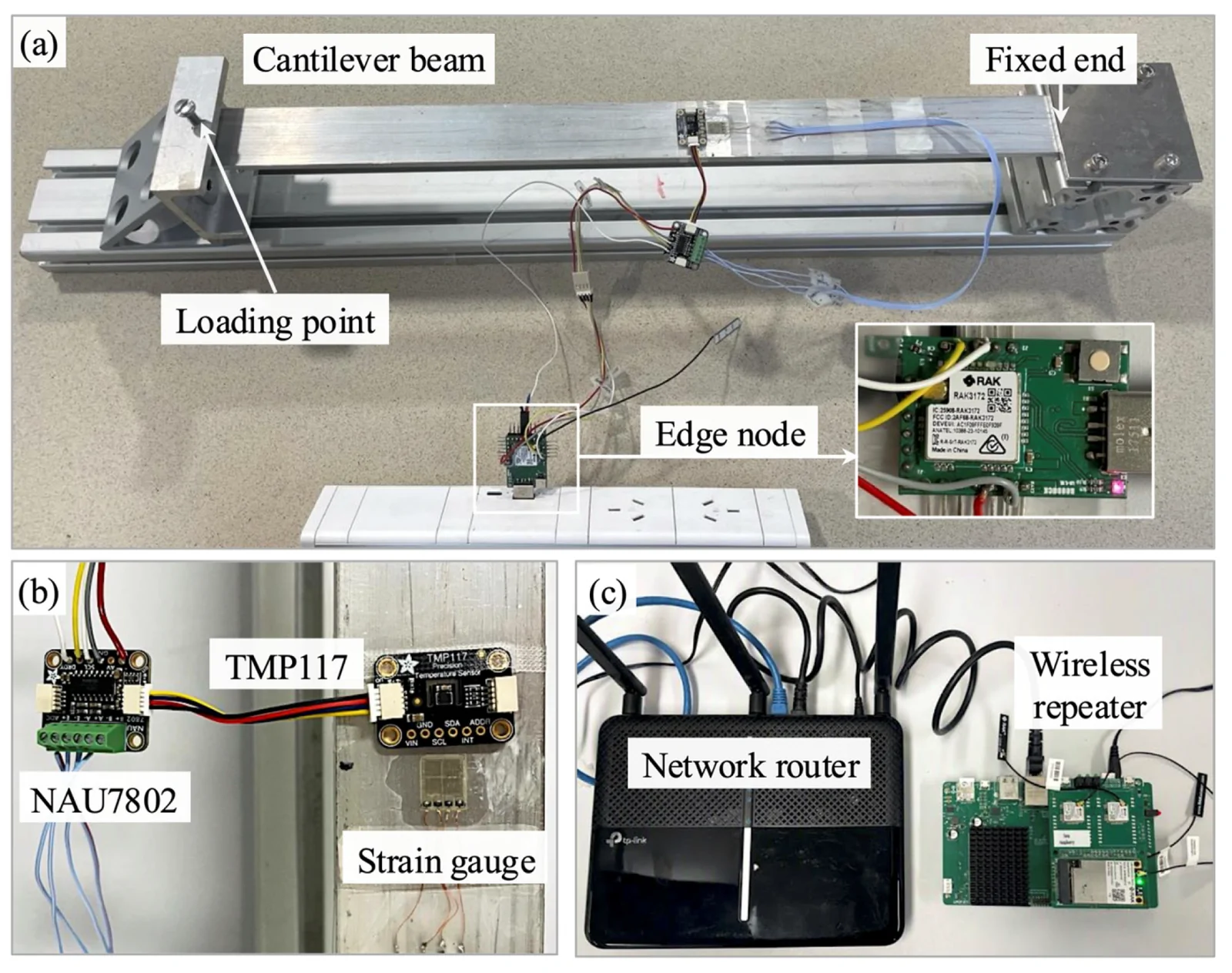
Laboratory evaluation setup: (**a**) cantilever fixture, loading point, and sensing node; (**b**) strain-gauge and temperature-sensing interface; and (**c**) LoRa–MQTT gateway and surface application.

The loading screw applied downward deflection to the aluminum cantilever (Figure 2), producing longitudinal bending at the instrumented section. The loading span was 512.2 mm, the beam thickness was 6.0 mm, and the center of the strain gauge was 210.0 mm from the fixed end. The M6 loading screw had a nominal pitch of 1.0 mm per complete 360° revolution. This step-loading procedure was repeated in ten runs, during which the run-median temperature estimates ranged from 10.6 to 33.1 °C. These descriptive temperature estimates were derived from a separately recorded ambient-temperature series mapped to the TMP117 scale using a pre-established linear calibration; they were not used in the loading regressions.

At the start of every run, the screw was advanced nominally from 0 to 1 mm to establish contact with the cantilever loading surface. This initial contact step was excluded from the analysis, which covered nominal screw advances from 2 to 11 mm in 1 mm increments. Acquisition at each setting began after a 5 s delay, after which 100 bridge-count summaries were recorded at nominal 0.5 s intervals. Each summary averaged five valid samples. Across runs, the observed run-median intervals ranged from 0.508 s to 0.511 s, or approximately 2 Hz. These diagnostic summaries were used only for the loading analysis and were separate from the 1 s telemetry records.

Outlier screening was performed separately within each run and nominal screw position on the diagnostic bridge-count summaries. This process employed the absolute modified Z-score based on the median absolute deviation (MAD) [30]. A summary was excluded when the absolute modified Z-score exceeded 3.5. If the scaled MAD was zero, all finite summaries were retained. The retained set provided the level mean and sample standard deviation.

Regression inputs were not baseline shifted. Each run was analyzed by unweighted ordinary least-squares regression using the unshifted mean bridge count at each screw setting (“level mean”) and a freely estimated intercept. The reported diagnostics were the coefficient of determination (*R*^2^), root-mean-square error, and maximum absolute residual. The ten fitted slopes were summarized by their mean, sample SD, and coefficient of variation (CV). For display only, the retained 2 mm mean was subtracted from the plotted counts. Figure error bars denote within-setting sample standard deviations, and annotations denote run-median temperature estimates. MATLAB R2025b (The MathWorks, Inc., Natick, MA, USA) was used for the analysis. The repeated fixture-loading results are presented in Section 3.1 and Supplementary Table S1.

#### 2.6.2. Fixed-Setting Natural-Temperature Test Procedure

Fixed-setting natural-temperature evaluations were conducted with the loading screw maintained at the nominal 11 mm setting. The same cantilever configuration, embedded compensation equation, and coefficient set were used consistently across evaluations. The fixture was positioned outdoors in the shade, allowing the TMP117 to record natural temperature variations without active environmental control. For each evaluation, records within the initial 120 s were excluded to omit initialization, configuration, and taring. All subsequent valid telemetry records were retained for the primary analysis. The fixed screw setting held the nominal mechanical configuration constant; it was not an independent verification that strain or load remained constant during temperature variation.

For each evaluation, apparent thermal sensitivity was quantified by an ordinary least-squares fit of apparent strain against measured temperature over the complete retained dataset. For descriptive visualization, the retained records were additionally summarized in non-overlapping 60 s windows as described in Supplementary Methods S1; these window means were used only as plotted markers and were not used to estimate the reported slopes or percentage reductions. The evaluation results are presented in Section 3.2, and the descriptive responses are shown in Supplementary Figure S1.

#### 2.6.3. Integrated Functional Evaluation

Each upper-limit, positive-spike, and absolute-rate rule was tested in 15 operator-controlled trials through the full node-to-dashboard communication path. Before the dashboard response was inspected, the onset condition and expected trigger or non-trigger outcome were recorded. The trials included below- and above-threshold profiles. The absolute-rate tests used a 5 min window and a threshold of 2 µε/min and included slowly varying, monotonic, stepped, and late-window profiles. The upper-limit tests used thresholds of 5, 10, 15, 50, 100, 150, 200, 240, and 340 µε. Positive-spike tests included gradual and rapid deflections designed to produce isolated and simultaneous event combinations.

The dashboard workflow included event decoding, audible alarms, manual clearance commands, and independent processing of event-specific acknowledgments. Strain inputs were applied manually, without actuator control or a synchronized dynamic load reference. Subjective operator influence on the imposed input profiles therefore cannot be excluded. Accordingly, these tests assessed rule execution and event handling under the recorded apparent-strain profiles; they did not quantify the repeatability of the mechanical input or the accuracy of the physical threshold crossing.

Separately, routine record delivery over the implemented LoRa-to-Node-RED path was characterized in three laboratory sessions. Generated-record totals were determined from the final source-sequence value in each session, and unique received records were counted from retained Node-RED captures. For each session, missing source-sequence positions, the maximum consecutive missing run, duplicate records, malformed or unusable records, out-of-order unique records, and receiver interarrival intervals based on host receive timestamps were determined. Received Signal Strength Indicator (RSSI), Signal-to-Noise Ratio (SNR), and hop-specific diagnostics were not recorded. Detailed session-level results are provided in Supplementary Table S5.

History reconstruction was tested in a planned matrix of 60 trials at the Node-RED application layer, after wireless transport and deduplication. A temporary fault-injection gate placed immediately before the history decoder altered selected incoming records in a controlled manner. The matrix comprised 10 no-fault 60-record baselines; 10 first-target-DATA omissions; 10 first-target-DATA payload-bit flips; 5 first-target-DATA duplications; 5 adjacent-DATA reorderings; 4 wrong-session first arrivals; 3 wrong-sequence first arrivals; 3 invalid entry-count headers; 5 odd 61-record transfers; and 5 near-capacity 1024-record circular-buffer wrap transfers. For each trial, an unchanged copy of the targeted incoming frame was retained for comparison. Only the first selected arrival was altered; later arrivals, including retransmissions, passed unchanged.

A trial passed only if the system detected the injected fault or the resulting sequence gap, reconstructed a complete record set identical to the reference copy, and passed CRC-32 verification. As part of the formal experimental audit, each observed BEGIN-declared record total was compared with the expected count in the trial manifest. This comparison was separate from the production decoder, whose sequence-continuity, END actual, and CRC checks assessed the internal consistency of the received transfer. For reporting the conditional protocol-evaluable subset, an attempt was included when the experimental transaction had started and the trial ended with a terminal PASS or FAIL classification. The remaining eight attempts were retained in the complete 60-attempt result but excluded from this conditional subset: two FAIL attempts that ended before the experimental transaction started, three INTERRUPTED attempts without terminal PASS or FAIL classifications, and three INVALID/CONTAMINATED attempts. Supplementary Tables S4 and S6 report the aggregate outcomes and the disposition of all ten non-pass attempts.

For the one-asset metadata check, the node was reset and the stored BOLT-001 record was queried over the wireless link. The surface application verified the CRC-32 checksum, decoded the location-metadata packet payload, and compared the identity and spatial fields with the laboratory registry. The check passed only when every compared field matched. Routine-telemetry, operator-controlled alarm, history-transfer, and metadata-matching results are presented in Section 3.3. Supplementary Tables S5 and S6 provide the detailed routine-telemetry and history-transfer audit results, respectively.

## 3. Results

### 3.1. Repeated Fixture-Level Loading Response

Following scaled MAD filtering, 9969 of the 10,000 diagnostic bridge-count summaries were retained. Across the ten runs, ordinary least-squares fits from 2 mm to 11 mm had *R*^2^ > 0.9997, with slopes of (59.94–60.80) × 10^3^ counts/mm. The slopes averaged (60.31 ± 0.33) × 10^3^ counts/mm, corresponding to a coefficient of variation of 0.55%. These statistics characterize the repeatability of the bridge-count response to nominal screw advance within the tested loading procedure. The run-median temperature estimates were used only for descriptive cross-run comparison and were not controlled as experimental set points. Run-specific root-mean-square errors were (0.32–2.68) × 10^3^ counts, and the maximum absolute level-mean residual was 5.58 × 10^3^ counts (1.03% of the associated run’s level-mean count span). Detailed run-level retention, fit, and residual statistics are provided in Supplementary Table S1.

**Figure 3:**
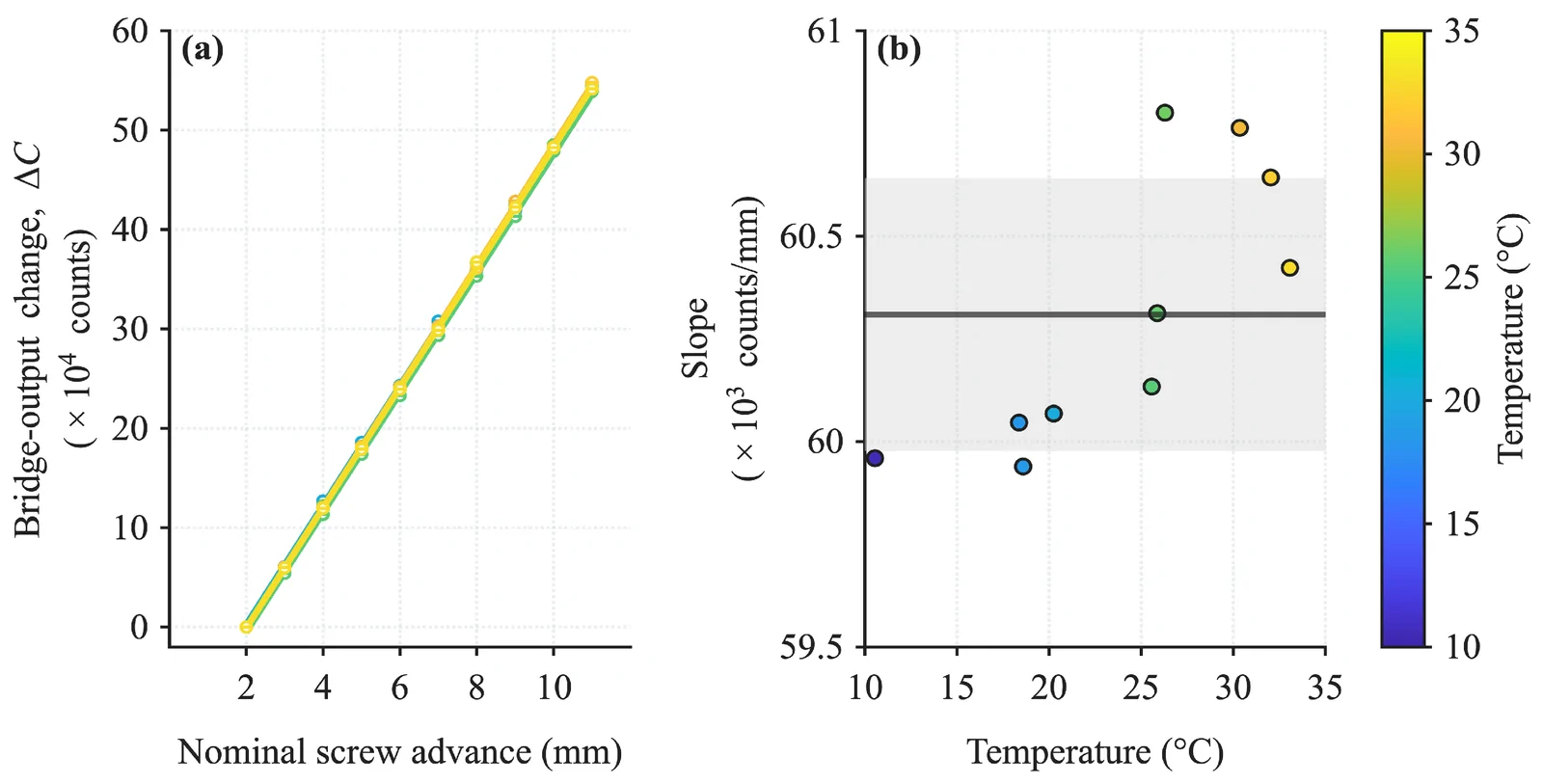
Repeated fixture-loading responses: (**a**) bridge-count change versus nominal screw advance for ten runs; and (**b**) run-wise fitted slope versus run-median temperature estimate.

For display in Figure 3a, the retained mean at the nominal 2 mm setting was subtracted within each run; this setting was not treated as zero load. Figure 3b pairs each fitted slope with the corresponding run-median temperature estimate.

### 3.2. Fixed-Setting Natural-Temperature Evaluations

The three fixed-setting evaluations are summarized in Table 2. Across temperature spans of 5.06, 7.99, and 5.76 °C, the unchanged embedded compensation reduced the magnitude of fitted apparent thermal sensitivity by 90.6%, 95.5%, and 89.1%, respectively. The corresponding apparent-strain responses are shown in Supplementary Figure S1.

### 3.3. Routine Telemetry, Alarms, History Retrieval, and Metadata Matching

Across three routine-telemetry sessions, 3415 of 3446 generated records were uniquely received, corresponding to an aggregate record-delivery ratio of 99.10% (session range, 98.87–99.30%). The maximum consecutive missing runs were 2, 1, and 4 records. The corresponding maximum receiver interarrival intervals, calculated from Node-RED host receive timestamps, were 3.013, 2.007, and 4.921 s, respectively; these intervals describe receiver-side arrival spacing and are not end-to-end latency measurements. No duplicate records, malformed or unusable records, or out-of-order unique records were observed in the retained captures. Further details are provided in Supplementary Table S5.

Across 45 operator-controlled laboratory trials (15 per rule), the observed trigger or non-trigger outcomes matched the expectations recorded before dashboard inspection. Triggered trials generated LoRa-transmitted event records and latched dashboard states. With the upper-limit rule set to 10 µε and 2 µε hysteresis, the first sampled values after manual threshold crossings were 13.76 µε during activation and 5.57 µε during reset. Because the screw crossed the boundaries between samples, these values are observed post-crossing samples, not estimates of the exact transition thresholds. Positive-spike trials produced isolated or concurrent rule outcomes according to the recorded profiles. For the 5 min absolute-rate rule, an approximately 1 µε/min drift remained below the 2 µε/min threshold, whereas monotonic, stepped, and late-window profiles above the threshold generated rate events.

Of the 60 planned history-transfer attempts, 50 passed, four failed, three were interrupted, and three were invalid or contaminated, giving an overall pass fraction of 50/60. The conditional protocol-evaluable subset included only attempts in which the experimental transaction had started and a terminal PASS or FAIL classification was recorded. Two of the four FAIL attempts, BASE-06 and WRAP1024-03, ended before the experimental transaction started and were therefore excluded. The conditional subset consequently comprised 50 PASS and 2 FAIL attempts (52 in total); 50 of these 52 attempts passed. All 38 fault-injection trials within this subset passed. The two FAIL outcomes within the conditional protocol-evaluable subset occurred during no-fault baseline transfers. For BASE-01 and BASE-04, the observed BEGIN totals were 793 and 27 records, respectively, although each trial requested a 60-record range. For BASE-01, the anomalous BEGIN declaration was identified in the retained audit stream and the operator stopped the transfer. For BASE-04, the experiment runner detected the manifest mismatch and issued FETCH STOP. Neither attempt reached CRC verification nor produced a completed, CRC-verified history output. Successful tests included exact reconstruction of a 1024-record circular-buffer transfer. Supplementary Results S2 and Table S6 provide the disposition of all ten non-pass attempts.

After resetting the node, a wireless query returned the stored BOLT-001 metadata. The returned location-metadata packet payload passed CRC-32 verification, and every decoded field matched the laboratory registry (Table 3).

## 4. Discussion

### 4.1. Principal Findings and Measurement Interpretation

Taken together, the laboratory tests show that the prototype combined measurement processing, local alarm evaluation, history recovery, and single-asset metadata matching within one edge-to-surface workflow. The findings support the operation of this integrated workflow under the tested conditions, while absolute strain accuracy, underground wireless performance, and multi-asset operation remain outside the present evidence.

The repeated loading experiments produced a highly linear bridge-count response to nominal screw advance, with only 0.55% variation in fitted slope across the ten runs. This result indicates a consistent fixture-level bridge-count response within the tested loading procedure. The fixed coefficient was retained only as the implemented apparent-strain scale used in the subsequent evaluations; the repeated-loading result does not independently validate its magnitude. Because no independent strain, displacement, or load reference was used, the converted values represent tare-referenced apparent strain rather than traceable absolute strain.

Temperature compensation reduced temperature-correlated apparent strain in all three fixed-setting evaluations. With the same embedded equation and unchanged coefficient set, the magnitude of the fitted apparent thermal sensitivity decreased by 90.6%, 95.5%, and 89.1% in Evaluations 1–3, respectively. The reductions observed across three natural-temperature records support repeated improvement within the tested cantilever configuration without re-estimating the coefficients. Residual structure nevertheless remained visible in the descriptive plots, so the reported percentages quantify reductions in fitted temperature sensitivity rather than the complete removal of all temperature-related variation. Generality across sensor assemblies, substrates, installation conditions, and thermal environments remains to be evaluated; the coefficients require revalidation whenever any of these factors changes.

The 45 operator-controlled alarm trials showed that the three rules produced the expected trigger or non-trigger outcomes under the recorded apparent-strain profiles and that the resulting event records were transmitted and handled by the surface application. The trials therefore support the implemented rule logic and event-handling workflow. Quantitative evaluation of threshold-crossing accuracy and mechanical repeatability will require actuator-controlled loading and an independent dynamic reference.

### 4.2. Operational Significance and Relation to Prior Work

The laboratory results illustrate how each interface preserves the meaning of a monitoring record from the edge node to the surface application. Local temperature compensation reduces temperature-related variation before transmission, and local alarm evaluation allows the node to identify an event without waiting for the surface application to perform the calculation. Routine summaries describe current measurements, buffered history can restore context for earlier events or missing intervals, and persistent metadata links each record to the monitored support. These functions address different points at which information can be lost or misinterpreted in the monitoring chain.

Building on prior studies of individual sensing, temperature-compensation, edge-processing, and wireless-monitoring functions [9,10,16,19,20,21], the present study jointly evaluates their interfaces within an integrated laboratory system. The contribution therefore lies primarily in system integration and interface-level evaluation.

The controlled cantilever fixture served as an intermediate test platform before integration into a packaged hollow rock bolt. It allowed the measurement, compensation, alarm, and communication functions to be examined under controlled laboratory conditions before introducing the additional mechanical and installation variables of a packaged bolt. The results therefore describe the implemented laboratory system; bolt-scale mechanical behavior requires direct validation.

### 4.3. Communication Recovery and Metadata Matching

Across the three routine-telemetry sessions, 99.10% of generated records were uniquely received by the surface application. This result shows that the implemented LoRa-to-Node-RED path supported approximately 1 Hz reporting with only short missing-record sequences under the tested laboratory conditions. The result therefore characterizes laboratory link performance only; underground propagation requires separate field evaluation.

The two pass fractions reported in Section 3.3 describe different populations. The 50/60 result preserves the disposition of the complete planned test set, whereas 50/52 describes the observed outcome of the conditional protocol-evaluable subset, defined by attempts in which the experimental transaction had started and a terminal PASS or FAIL classification was recorded. Neither fraction measures end-to-end nor physical wireless-channel reliability. Successful recovery from the planned record alterations, together with complete reconstruction of the 1024-record circular-buffer transfer, supports the decoder and retransmission workflow under the tested post-transport conditions.

After one node reset, the stored metadata for BOLT-001 remained retrievable, passed CRC-32 verification, and matched the laboratory registry. This single observation shows persistence and correct retrieval for one registered asset, but it does not establish the reliability of post-reset metadata retrieval across repeated resets. Multi-asset registration, identification, reassignment, identity-conflict handling, and registry scaling also remain to be evaluated.

### 4.4. Applicability to Underground Deployment

The laboratory tests show that the monitoring workflow operated under the tested fixture and radio conditions. Underground deployment will add mechanical, thermal, and communication variability. Radio propagation may be affected by roadway geometry and curvature, junctions, machinery and moving obstructions, antenna position and enclosure, multipath propagation, and transitions between line-of-sight and non-line-of-sight conditions [12,13,14,15]. Fault injection was applied after wireless transport and therefore did not reproduce these underground propagation effects.

These conditions may increase packet loss, lengthen missing-record sequences, delay the arrival of event records at the surface, and increase retransmission demand. A sufficiently long interruption could exceed the finite circular-buffer capacity and reduce the amount of recent stored records available for recovery. Alarm evaluation would continue locally, but delivery of the event record and its historical context would depend on the underground link. Site-specific radio measurements and component-level link diagnostics are therefore needed to quantify these effects.

## 5. Conclusions

This study evaluated whether strain processing, local decision making, wireless telemetry, data recovery, and single-asset metadata matching could operate together within an integrated IoT monitoring workflow. The cantilever experiments demonstrated repeatable fixture-level bridge-count responses. Three fixed-setting natural-temperature evaluations showed substantial reductions in temperature-correlated apparent strain without re-estimating the compensation coefficients. Under operator-controlled inputs, the implemented alarm rules and event-delivery workflow produced outcomes that matched the expectations recorded before dashboard inspection. Routine LoRa sessions characterized record delivery only under laboratory radio conditions, whereas separate post-transport fault-injection tests assessed application-layer history reconstruction through sequence continuity and CRC-32 verification. After node reset, one stored asset record was retrieved and matched to the laboratory registry.

Taken together, the results demonstrate an integrated laboratory workflow linking measurement, alarm, recent stored records, and asset identity and location from the edge node to the surface application. This integration matters because a surface user must be able to interpret a measurement, recover the context surrounding an event or missing interval, and associate the record with the monitored support. Bolt-scale mechanical accuracy, underground loss recovery, and multi-asset reliability remain to be validated. Underground geometry, obstructions, and intermittent links may increase packet loss, latency, and retransmission demand; a sufficiently long interruption may exceed the finite circular-buffer capacity and reduce the recent history available for recovery. The next validation stage should therefore compare a packaged hollow-bolt prototype against independent mechanical references, perform controlled thermal cycling over wider temperature ranges and longer durations, repeat actuator-controlled alarm tests after integration, and quantify physical-channel loss, RSSI, SNR, burst loss, latency, retransmission, buffer sufficiency, and registry behavior in a multi-asset underground deployment.

## Figures and Tables

**Table 1:** Hardware, software, and tested settings of the laboratory system.

System Stage	Component	Evaluated Configuration
Sensing interface	BF1000-3EB 1000 Ω full-bridge foil strain gauge	2.4 V excitation; approximately 2.36 mA
	Adafruit TMP117 QT temperature-sensor board	Local temperature measurement; default device configuration
	Adafruit NAU7802 QT 24-bit ADC board	Channel 1; 2.4 V LDO; PGA gain 128; 10 SPS
Edge node	RAK3172 module incorporating an STM32WLE5 microcontroller	10 Hz sample processing; approximately 1 Hz routine reporting; 1024-record circular buffer
Wireless link	LoRa radio link	Laboratory evaluation; 926.0 MHz uplink; 921.0 MHz downlink; 22 dBm transmit power; SF7; CR 4/5; 500 kHz bandwidth; 8-symbol preamble; PHY CRC enabled
Surface gateway and application	Custom CM4-based LoRa repeater and Node-RED v4.1.0 surface application	Repeater software v0.8.2; MQTT protocol version 3.1.1; record parsing and logging; CRC-32 verification; history reconstruction; registry comparison

Abbreviations: ADC, analog-to-digital converter; CR, coding rate; CRC, cyclic redundancy check; LDO, low-dropout regulator; MQTT, Message Queuing Telemetry Transport; PGA, programmable gain amplifier; PHY, physical layer; SF, spreading factor; SPS, samples per second.

**Table 2:** Fixed-setting natural-temperature evaluations using the unchanged embedded compensation parameters.

Evaluation	Duration (h)	TMP117 Range (°C)	Δ*T* (°C)	Uncompensated Slope (µε/°C)	Embedded Slope (µε/°C)	Reduction (%)
1	8.19	17.30–22.36	5.06	+1.665	+0.156	90.6
2	9.47	13.52–21.51	7.99	+1.656	−0.075	95.5
3	10.45	14.39–20.15	5.76	+1.815	+0.198	89.1

Note: Slopes are ordinary least-squares estimates of apparent strain versus TMP117 temperature. Fitted signs are retained. Reduction was calculated from the absolute unrounded slopes.

**Table 3:** Comparison of registered and decoded fields from the 78-byte location-metadata packet for BOLT-001.

Field	Registered Value	Radio-Decoded Value	Match
Asset identifier	BOLT-001	BOLT-001	Yes
Location code	Z01-C100-R01-H01	Z01-C100-R01-H01	Yes
Map version	2	2	Yes
Laboratory site/rig IDs	1/100	1/100	Yes
Chainage/row/hole	210 mm/1/1	210 mm/1/1	Yes
Fixture coordinates	(210, 0, 0) mm	(210, 0, 0) mm	Yes
Coordinate-reference ID	1	1	Yes
Installation timestamp	1,774,382,043	1,774,382,043	Yes
Material/calibration IDs	1/1	1/1	Yes

Note: Mine identifier 1 and roadway identifier 100 are laboratory registry codes; coordinates are expressed in the cantilever-fixture reference frame. These registry coordinates are metadata fields and are distinct from the strain-gauge position used in the cantilever description. The received packet used schema 1 and had a verified CRC-32 value of 0xF06C479F (4,033,628,063 in decimal).

## Data Availability

The retained data underlying the analyses reported in this study, together with the analysis scripts used to verify the reported metrics, are available from the corresponding author on reasonable request.

## Supplementary Materials

The following supporting information can be downloaded at: https://www.mdpi.com/article/10.3390/s26185686/s1, Table S1, Run-level statistics for ten repeated fixture-loading experiments (provided as a separate editable spreadsheet); Supplementary Methods S1, Temperature-Sensitivity Analysis and Descriptive Visualization; Supplementary Results S1, Fixed-Setting Natural-Temperature Responses; Figure S1, Apparent-strain responses in the three fixed-setting natural-temperature evaluations; Supplementary Methods S2, Selection of Embedded Temperature-Compensation Parameters; Supplementary Methods S3, Binary History and Location Packet Layouts; Table S2, Complete byte layout of history-transfer format and its 32-byte entry; Table S3, Complete byte layout of the 78-byte LOCATION_V1 packet; Supplementary Methods S4, Planned History-Transfer Fault-Injection Matrix; Supplementary Results S2, History-Transfer Fault-Injection Outcomes; Table S4, Planned conditions and final outcomes for the 60-trial history-transfer matrix; Table S5, Record-level delivery observed during three routine-telemetry sessions (provided as a separate editable spreadsheet); Table S6, Trial-level disposition and evidence basis for all ten non-pass history-transfer attempts (provided as a separate editable spreadsheet).
