# Supplementary material for: Evaluation of an IoT Strain-Sensing System with LoRa Telemetry and Asset Metadata
Source: Sensors (Basel). 2026 Sep 8;26(18):5686. doi: 10.3390/s26185686 (PMC13611494; doi:10.3390/s26185686)
Supplement: Supplementary file 1 [file sensors-26-05686-s001.zip › sensors-4501274-supplementary.pdf]

MDPI

sensors

Supplementary Materials

Evaluation of an IoT Strain-Sensing System with LoRa Telemetry and Asset Metadata

Xiaoxiao Bu [1], Shenshi Jiang [2], Henry Gong [3], Tommy Chaung [3], Enbang Li [2,*] and Zhengyi Jiang [1,*]

[1] School of Engineering, University of Wollongong, Wollongong, NSW 2522, Australia; xb376@uowmail.edu.au
[2] School of Mathematics and Physics, University of Wollongong, Wollongong, NSW 2522, Australia; sj863@uowmail.edu.au
[3] Roobuck Pty Ltd., Brookvale, NSW 2100, Australia; henryg@roobuck.com.au (H.G.); tommyc@roobuck.com.au (T.C.)
* **Correspondence:** enbang@uow.edu.au (E.L.); jiang@uow.edu.au (Z.J.)

This supplementary document contains Supplementary Methods S1–S4, Supplementary Results S1–S2, Tables S2–S4, and Figure S1. Supplementary Tables S1, S5, and S6 are provided as separate editable spreadsheets. Supplementary Table S1 provides run-level statistics for the repeated fixture-loading experiments; Supplementary Table S5 reports session-level routine-telemetry delivery metrics; and Supplementary Table S6 provides trial-level dispositions for the ten non-pass history-transfer attempts.

Supplementary Methods S1. Temperature-Sensitivity Analysis and Descriptive Visualization

For all three fixed-setting evaluations, the first 120 s were excluded and all subsequent valid telemetry records were retained for the analysis described in Section 2.6.2. Uncompensated and embedded-compensated apparent thermal sensitivities were estimated separately by ordinary least-squares regression against measured TMP117 temperature. Percentage reduction was calculated from the absolute unrounded fitted slopes.

For descriptive visualization only, the retained records were additionally summarized into non-overlapping 60 s windows using their timestamps. A plotted window was retained when it contained at least 30 valid whole-second observations. For each retained window, the mean TMP117 temperature and the corresponding mean uncompensated and embedded-compensated apparent strains were calculated. These window means were used only as graphical markers; the fitted slopes and reported percentage reductions were calculated from all retained telemetry records and were not re-estimated from the window means. All calculations were performed in MATLAB R2025b.

Supplementary Results S1. Fixed-Setting Natural-Temperature Responses

Across the three fixed-setting evaluations, embedded compensation reduced the magnitude of fitted apparent thermal sensitivity by 90.6%, 95.5%, and 89.1%, respectively. Figure S1 provides a descriptive visualization using non-overlapping 60 s window means. Residual variation remained visible after compensation, so the reported percentages quantify reduction in fitted temperature sensitivity rather than removal of all temperature-related apparent-strain variation.

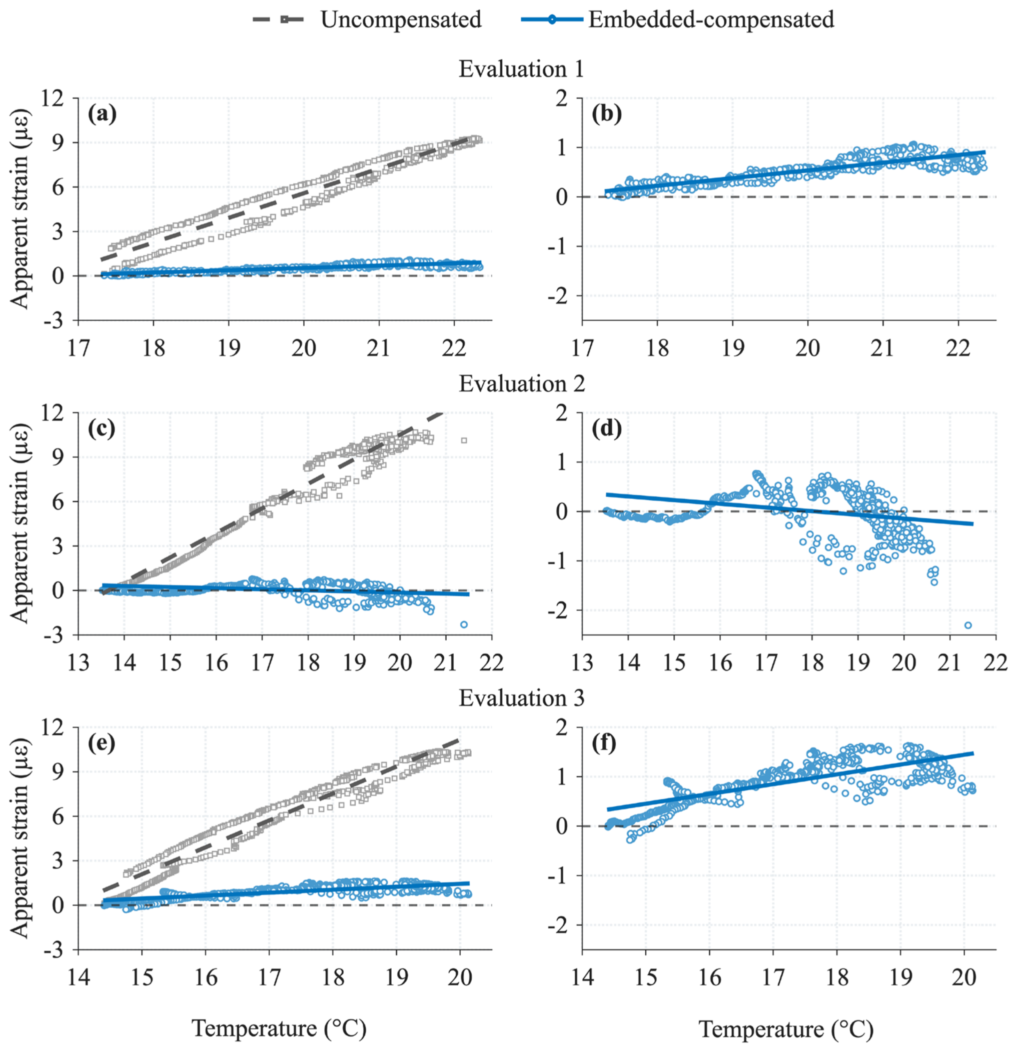

Figure S1. Apparent-strain responses in the three fixed-setting natural-temperature evaluations. Panels **(a)**, **(c)**, and **(e)** compare uncompensated and embedded-compensated apparent strain against TMP117 temperature for Evaluations 1–3, respectively; panels **(b)**, **(d)**, and **(f)** show the corresponding embedded-compensated responses on an expanded vertical scale. Markers are descriptive means from non-overlapping 60 s windows, whereas the straight lines are ordinary least-squares fits to all retained telemetry records.

Supplementary Methods S2. Selection of Embedded Temperature-Compensation Parameters

The parameter-selection process used repeated preliminary fixed-setting sessions conducted on non-consecutive days. One archived session retained as part of the coefficient-selection record contained 24,210 telemetry records collected at approximately 1 s intervals and spanned 17.95–26.64 °C. Candidate coefficient sets were evaluated iteratively while τ was held at 8385.1 s. Four (a_1, a_2, a_3) triplets were retained in the parameter-selection record: (3.1465, −0.12125, 1.7238), (2.9577, −0.15035, 1.7066), (2.3662, −0.12028, 1.3653), and (2.2921, −0.11650, 1.3226); the corresponding units were με/°C, με/°C², and με/°C.

The fourth triplet was selected from the archived preliminary sessions by visual inspection of residual patterns. The selection was not based on a prespecified optimization criterion; the candidates are therefore reported to document a configuration-specific engineering choice, not a ranked statistical comparison.

The final coefficient set used τ_{heat} = τ_{cool} = 8385.1 s together with the fourth (a_1, a_2, a_3) triplet. It was applied unchanged in all three fixed-setting evaluations; heating/cooling

state did not select branch-specific parameter values, and none of the evaluation datasets was used to re-estimate the parameters.

Supplementary Methods S3. Binary History and Location Packet Layouts

The evaluated history-transfer format is summarized in Table S2. Multi-byte integers were encoded in little-endian format, and floating-point quantities used IEEE 754 binary32 representation. Each transfer consisted of `BEGIN`, `DATA`, and `END` packets, with each `DATA` packet carrying up to two 32-byte history entries. The `END` packet contained a cyclic redundancy check (CRC) value calculated using the CRC-32/ISO-HDLC parameter set (polynomial `0x04C11DB7`; reflected implementation `0xEDB88320`; initial value `0xFFFFFFFF`; final exclusive-OR value `0xFFFFFFFF`; reflected input and output). The CRC covered the concatenated history-entry bytes from all sequenced `DATA` packets and excluded the packet headers and the `BEGIN` and `END` packets. After sequence and CRC verification, the surface application issued an `ASCII ACK` command. Missing `DATA` packets were requested using `NAK` commands. The space-separated `session` identifier and zero-based `sequence` number were transmitted as unsigned 16-bit decimal values. A valid `NAK` initiated three retransmissions of the specified `DATA` packet at 300 ms intervals.

Table S2. Complete byte layout of history-transfer format and its 32-byte entry.

Packet / byte offset(s)	Field	Encoding	Definition
`BEGIN 0`	`packet_type`	`uint8; 0x00`	Identifies a `BEGIN` packet.
`BEGIN 1–2`	`session`	`uint16, LE`	Transfer-`session` identifier.
`BEGIN 3–4`	`total`	`uint16, LE`	Number of history entries requested for the transfer.
`BEGIN 5`	`rate`	`uint8`	Nominal record `rate`; 1 denotes 1 Hz.
`BEGIN 6–9`	`start_sec`	`uint32, LE`	Monotonic second index of the oldest selected entry.
`DATA 0`	`packet_type`	`uint8; 0x01`	Identifies a `DATA` packet.
`DATA 1–2`	`session`	`uint16, LE`	Transfer-`session` identifier.
`DATA 3–4`	`sequence`	`uint16, LE`	Zero-based `DATA`-packet `sequence` number.
`DATA 5`	`count`	`uint8`	Number of entries in this packet (1 or 2).
`DATA 6–37`	`entry 0`	`entry (32 bytes)`	First history entry.
`DATA 38–69`	`entry 1`	`entry (32 bytes)`	Second entry when `count` = 2; absent when `count` = 1.
`END 0`	`packet_type`	`uint8; 0x02`	Identifies an `END` packet.
`END 1–2`	`session`	`uint16, LE`	Transfer-`session` identifier.
`END 3–4`	`actual`	`uint16, LE`	Number of entries transmitted.
`END 5–8`	`crc32`	`uint32, LE`	CRC-32/ISO-HDLC over concatenated `DATA`-entry bytes.

Packet / byte offset(s)	Field	Encoding	Definition
Entry 0–3	raw_mean	int32, LE	Mean bridge count in the record covering 1 s (counts).
Entry 4–7	ue_c_mean	float32, LE	Mean compensated apparent strain (με).
Entry 8–11	ue_c_min	float32, LE	Minimum compensated apparent strain during the record (με).
Entry 12–15	ue_c_max	float32, LE	Maximum compensated apparent strain during the record (με).
Entry 16–19	max_rate	float32, LE	Maximum absolute adjacent-sample apparent-strain rate, $\lvert\Delta\varepsilon/\Delta t\rvert$, within the 1 s history record (με/s); this per-record diagnostic is separate from the configurable alarm-rate calculation in Section 2.4.
Entry 20–23	max_spike	float32, LE	Maximum absolute adjacent-sample apparent-strain change, $\lvert\Delta\varepsilon\rvert$, within the 1 s history record (με); this per-record diagnostic is separate from the positive-spike alarm rule in Section 2.4.
Entry 24–25	temp_x100	int16, LE	Temperature stored in 0.01 °C units; physical temperature = (stored value/100) °C.
Entry 26	alarm	uint8	Sticky alarm flag for the record covering 1 s (0 or 1).
Entry 27	reserved	uint8; 0	Reserved byte.
Entry 28–31	sec_idx	uint32, LE	Monotonic index of records at 1 s resolution.

As detailed in Table S3, LOCATION_V1 consisted of a fixed 78-byte message. Numeric fields were little-endian, and the two fixed-width text fields were ASCII-encoded and NUL-padded. Its CRC used the same CRC-32/ISO-HDLC parameters and covered bytes 0–73. The registered value installation_time = 1,774,382,043 corresponded to 2026-03-25 06:54:03 AEDT (UTC+11) when converted from Unix time.

Table S3. Complete byte layout of the 78-byte LOCATION_V1 packet.

Byte offset(s)	Field	Encoding	Definition
0	packet_type	uint8; 0xA1	Identifies a LOCATION_V1 packet.
1	schema_version	uint8; 1	Location-schema version.

2	flags	uint8 bit mask	Bit 0: identity configured; bit 1: location code configured; bit 2: coordinates configured; bits 3–7: reserved and set to 0.
3	reserved	uint8; 0	Reserved byte.
4-19	bolt_id	ASCII (16 bytes)	NUL-padded registered asset identifier.
20-35	loc_code	ASCII (16 bytes)	NUL-padded registered location code.
36-39	map_version	uint32, LE	Map or registry version.
40-41	mine_id	uint16, LE	Mine/site registry code.
42-43	roadway_id	uint16, LE	Roadway/fixture registry code.
44-47	chainage_mm	int32, LE	Signed chainage coordinate (mm).
48-49	row_no	uint16, LE	Registered row number.
50-51	hole_no	uint16, LE	Registered hole number.
52-55	x_mm	int32, LE	Signed local x coordinate (mm).
56-59	y_mm	int32, LE	Signed local y coordinate (mm).
60-63	z_mm	int32, LE	Signed local z coordinate (mm).
64-65	coordinate_reference_id	uint16, LE	Coordinate-reference registry identifier.
66-69	installation_time	uint32, LE	Registry-supplied uint32 timestamp; the laboratory registry used Unix seconds from the UTC epoch.
70-71	material_id	uint16, LE	Material registry identifier.
72-73	calibration_id	uint16, LE	Calibration/configuration registry identifier.
74-77	crc32	uint32, LE	CRC-32/ISO-HDLC over bytes 0–73.

Abbreviations: LE, little-endian; NUL, zero-valued padding byte. Firmware field names are retained to document the transmitted binary schema.

Supplementary Methods S4. Planned History-Transfer Fault-Injection Matrix

The history-transfer protocol was evaluated using a 60-trial matrix defined before testing. A temporary fault-injection gate was inserted in the Node-RED application stream after transport deduplication and immediately before the history decoder, so controlled changes were applied at the application layer after wireless transport. These trials therefore evaluated decoder and recovery behavior rather than physical-channel effects. For fault-injection trials, an unchanged copy of the targeted incoming DATA packet was retained as a reference, and only the first targeted arrival was modified; subsequent arrivals, including retransmissions, passed unchanged. The matrix covered no-fault 60-record baselines; first-target-DATA omission, payload-bit flip, duplication, adjacent-packet reordering, wrong-session, wrong-sequence, and invalid-entry-count-header conditions; odd 61-record transfers; and near-capacity 1024-record circular-buffer transfers. Requested counts and planned repetitions are summarized in Table S4.

A trial passed when the planned change was applied where applicable, the resulting inconsistency was detected, the transfer completed with zero missing records, the reconstructed fields matched the reference copy, and CRC-32 verification succeeded. For reporting the conditional protocol-evaluable subset, an attempt was included when the experimental transaction had started and the trial ended with a terminal `PASS` or `FAIL` classification. This inclusion rule was applied to the final trial classifications; the 52 attempts therefore form a conditional subset rather than a pre-specified denominator. The remaining eight attempts comprised two `FAIL` attempts that ended before the experimental transaction started, three `INTERRUPTED` attempts without terminal `PASS` or `FAIL` classifications, and three attempts with incomplete required incoming records outside the planned fault injection. All eight attempts remained part of the complete 60-attempt result. Table S4 summarizes the final outcomes, and Table S6 provides the disposition of all ten non-pass attempts.

Supplementary Results S2. History-Transfer Fault-Injection Outcomes

Among all 60 planned attempts, 50 passed, 4 failed, 3 were interrupted, and 3 were classified as `INVALID/CONTAMINATED` (Table S4), giving an overall pass fraction of 50/60. The conditional protocol-evaluable subset comprised 52 attempts in which the experimental transaction had started and a terminal `PASS` or `FAIL` classification was recorded; 50 of these 52 attempts passed. The two failures within this subset were no-fault baseline trials. For `BASE-01` and `BASE-04`, `BEGIN` totals of 793 and 27 records, respectively, did not match the 60 records requested in the trial manifest. For `BASE-01`, the anomalous `BEGIN` declaration was identified in the retained audit stream and the operator stopped the transfer. For `BASE-04`, the experiment runner detected the manifest mismatch and issued `FETCH STOP`. Neither attempt reached CRC verification or produced a completed, CRC-verified history output. The remaining eight attempts comprised two failures before the experimental transaction started, three interruptions, and three invalid or contaminated attempts.

All 38 fault-injection trials included in the analysis passed. Across the 50 passed trials, 28 application-level logical retransmissions were recorded, and each completed transfer ended with zero missing records, agreement with the retained reference copy, and a valid CRC-32. The uncontaminated `WRAP1024-05` trial reconstructed all 1024 requested records with zero field mismatches and matching firmware and reconstructed CRC-32 values of `0x6A7630BA`.

The three attempts with incomplete incoming records were excluded from the conditional protocol-evaluable subset because the required inputs were not intact. Because the planned changes were introduced after wireless transport, the fault-injection results do not provide a measure of physical-channel packet loss. Trial-level details are provided in Table S6.

Table S4. Planned conditions and final outcomes for the 60-trial history-transfer matrix.

**Condition**	**Planned trials**	**Requested records**	**Final outcomes (P/F/I/X)**	**Conditional protocol-evaluable subset (n)**
No-fault baseline	10	60	6/3/1/0	8
First-target-`DATA` omission	10	60	8/0/1/1	8
Payload-bit flip	10	60	10/0/0/0	10
First-target-`DATA` duplication	5	60	5/0/0/0	5
Adjacent-`DATA` reordering	5	60	5/0/0/0	5
Wrong-session first arrival	4	60	4/0/0/0	4

Condition	Planned trials	Requested records	Final outcomes (P/F/I/X)	Conditional protocol-evaluable subset (n)
Wrong-sequence first arrival	3	60	3/0/0/0	3
Invalid entry-count header	3	60	3/0/0/0	3
Odd-record boundary	5	61	5/0/0/0	5
Near-capacity circular-buffer wrap	5	1024	1/1/1/2	1
Total	60	60, 61, or 1024	50/4/3/3	52

Note: P, passed; F, failed; I, interrupted; X, invalid or contaminated. Attempts in the conditional protocol-evaluable subset were those in which the experimental transaction had started and a terminal `PASS` or `FAIL` classification was recorded. The 52-attempt subset was defined by this rule during final aggregation; all 60 planned attempts were retained in the overall result.
